# The Combination of *Aquilaria sinensis (Lour.) Gilg* and *Aucklandia costus Falc.* Volatile Oils Exerts Antidepressant Effects in a CUMS-Induced Rat Model by Regulating the HPA Axis and Levels of Neurotransmitters

**DOI:** 10.3389/fphar.2020.614413

**Published:** 2021-02-24

**Authors:** Huiting Li, Yuanhui Li, Xiaofei Zhang, Guilin Ren, Liangfeng Wang, Jianzhe Li, Mengxue Wang, Tao Ren, Yi Zhao, Ming Yang, Xiaoying Huang

**Affiliations:** ^1^ College of Pharmacy, Chengdu University of traditional Chinese Medicine, Chengdu, China; ^2^ Ministry of Education, Key Laboratory of Modern Preparation of Traditional Chinese Medicine, Jiangxi University of Traditional Chinese Medicine, Nanchang, China; ^3^ College of Pharmacy, Shaanxi University of Chinese Medicine, Xianyang, China; ^4^ Southwest Medical University Affiliated Hospital of Traditional Chinese Medicine, Luzhou, China

**Keywords:** *Aquilaria sinensis (Lour.) Gilg*, *Aucklandia costus Falc.*, volatile oil, depression, chronic unpredictable mild stress, monoamine neurotransmitter, hypothalamic-pituitary- adrenal axis, cholinergic neurotransmitter

## Abstract

The *Aquilaria sinensis (Lour.) Gilg* (CX)–*Aucklandia costus Falc.* (MX) herbal pair is frequently used in traditional Chinese medicine prescriptions for treating depression. The volatile oil from CX and MX has been shown to have good pharmacological activities on the central nervous system, but its curative effect and mechanism in the treatment of depression are unclear. Therefore, the antidepressant effect of the volatile oil from CX–MX (CMVO) was studied in chronic unpredictable mild stress (CUMS) rats. The suppressive effects of CMVO (25, 50, 100 μL/kg) against CUMS-induced depression-like behavior were evaluated using the forced swimming test (FST), open field test (OFT) and sucrose preference test (SPT). The results showed that CMVO exhibited an antidepressant effect, reversed the decreased sugar preference in the SPT and prolongation of immobility time in the FST induced by CUMS, increased the average speed, time to enter the central area, total moving distance, and enhanced the willingness of rats to explore the environment in the OFT. Inhalational administration of CMVO decreased levels of adrenocorticotropic hormone and corticosterone in serum and the expression of corticotropin-releasing hormone mRNA in the hypothalamus, which indicated regulation of over-activation of the hypothalamic–pituitary–adrenal (HPA) axis. In addition, CMVO restored levels of 5-hydroxytryptamine (5-HT), dopamine, norepinephrine and acetylcholine in the hippocampus. The RT-PCR and immunohistochemistry results showed that CMVO up-regulated the expression of 5-HT_1A_ mRNA. This study demonstrated the antidepressant effect of CMVO in CUMS rats, which was possibly mediated via modulation of monoamine and cholinergic neurotransmitters and regulation of the HPA axis.

## Introduction

Depression is an emotional rhythm disorder that has significant and lasting depressed mood as the main symptom (Difrancesco et al., 2019). It may be accompanied by insomnia, addiction, neurodegenerative diseases and other complications (Chen et al., 2019), and it seriously affects the physical and mental health of patients. The incidence of depression is increasing year by year. Currently, about 15% of people in the world are suffering from depression (Global Burden of Disease Study 2013, Collaborators, 2015). The World Health Organization predicted that it will become the second leading cause of disability after heart disease by 2020 (Holden, 2000). Based on the monoamine neurotransmitter deficiency hypothesis, the commonly used antidepressants are mainly chemical medicines such as selective serotonin reuptake inhibitors or norepinephrine reuptake inhibitors. However, these medicines have shortcomings that include poor curative effect, long duration of treatment and recurrence after drug withdrawal, and adverse reactions such as sexual dysfunction, nausea, tremor and insomnia (Zhuo et al., 2020). Studies (Antunes et al., 2015; Li et al., 2020b) have shown that one-third of patients do not respond to initial treatment, and almost half have only a secondary response. Therefore, it is very meaningful to look for natural medicines with better curative effects and suitability. Many studies have shown that volatile oils from aromatic herbs are potential natural medicines for the treatment of depression.

Aromatic herbs mainly contain volatile components, which are considered by traditional Chinese medicine (TCM) to relieve anxiety and other complications such as insomnia caused by depression. Fragrant traditional Chinese herbs have historically been used to regulate emotion and relieve depressive symptoms (Chen et al., 2019; Li et al., 2020a). Recent studies have shown that the volatile oils of clove (Mehta et al., 2013), fennel (Perveen et al., 2017), Cang-ai compound (Chen et al., 2019) and other aromatic traditional Chinese herbs can exert antidepressant effects by regulating the levels of monoamine neurotransmitters and activity of the hypothalamic-pituitary-adrenal (HPA) axis, or by improving immune function. Similarly, aromatherapy is also widely used in Western countries, exemplified by the use of lavender volatile oil to treat depression (López et al., 2017). Compared with other chemical components, volatile components more easily pass through the blood–brain barrier, which is advantageous in the treatment of central nervous system diseases (Zheng et al., 2018). Aromatherapy is not only effective, but also provides a pleasant treatment experience. It has unique advantages and broad development prospects for depression and other emotion-related chronic diseases.

Depression has complex etiologies and complications, so drug combinations are often used for treatment to exploit synergistic effects, improve efficacy or reduce adverse reactions, such as the combination of fluoxetine and olanzapine (Brunner et al., 2014). There is a similar mode of drug use in TCM, the herbal pair, which is the simplest form of compatibility in traditional Chinese herbal medicine (Wang et al., 2019b). *Aquilaria sinensis (Lour.)* Gilg (Chen-Xiang, CX) and *Aucklandia costus Falc*. (Mu-Xiang, MX), an ancient and classic herbal pair, has been commonly used to treat Yu-syndrome (Depression and Anxiety) in many traditional prescriptions, such as Er-xiang Powder recorded in “Jiyang Compendium.” Traditional Chinese medicine believes that the main causes of depression are stagnation of liver qi and dysfunction of spleen in transportation. Clinically, herbs with the effects of invigorating the spleen and soothing the liver qi are mainly used to treat depression. CX has the effects of activating qi and relieving pain, warming the stomach and relieving vomiting. It has a long history of clinical application, and is often used as a sedative, analgesic and digestive aid (Tan et al., 2019) in TCM. MX has the effects of invigorating qi and relieving pain, invigorating the spleen and eliminating food. These two herbs relieve depression, indigestion and other symptoms by soothing the liver, regulating qi and recuperating the spleen. Many proprietary Chinese medicines that relieve anxiety and irritability and reduce appetite contain both CX and MX, such as Chen xiang shu yu tablets, Chen xiang shu qi pills and Chen xiang hua qi tablets. Studies have shown that the volatile oil is one of the main active components in these two herbs. It has been demonstrated that the volatile oil from CX exerts antidepressant effects, possibly by inhibition of corticotropin-releasing hormone (CRH) and hyperactivity of the HPA axis (Wang et al., 2018). Other studies have suggested that the volatile oil of MX has potential neuroprotective activities, due to the regulation of apoptotic pathways (Zhao et al., 2016). Its main active ingredient, dehydrocostus lactone, elicited protective effects against hippocampal oxygen-glucose deprivation/reoxygenation injury by inhibiting apoptosis (Zhao et al., 2018). Besides, MX volatile oil and its main components have been proved to have immunosuppressive (Na et al., 2013), gastrointestinal regulation (Dong et al., 2018), anti-inflammatory (Woo et al., 2019) and other pharmacological effects, while CX volatile oil has a variety of pharmacological activities, such as antioxidation (Wang et al., 2018a), sedation (Wang et al., 2017), and anti-inflammation (Gao et al., 2019). However, the mechanism of CMVO in the treatment of depression is still unclear. Thus, it is of great significance to unravel its fundamental mechanisms, which may provide a novel and effective medicine and therapeutic method for depression.

**FIGURE 1:**
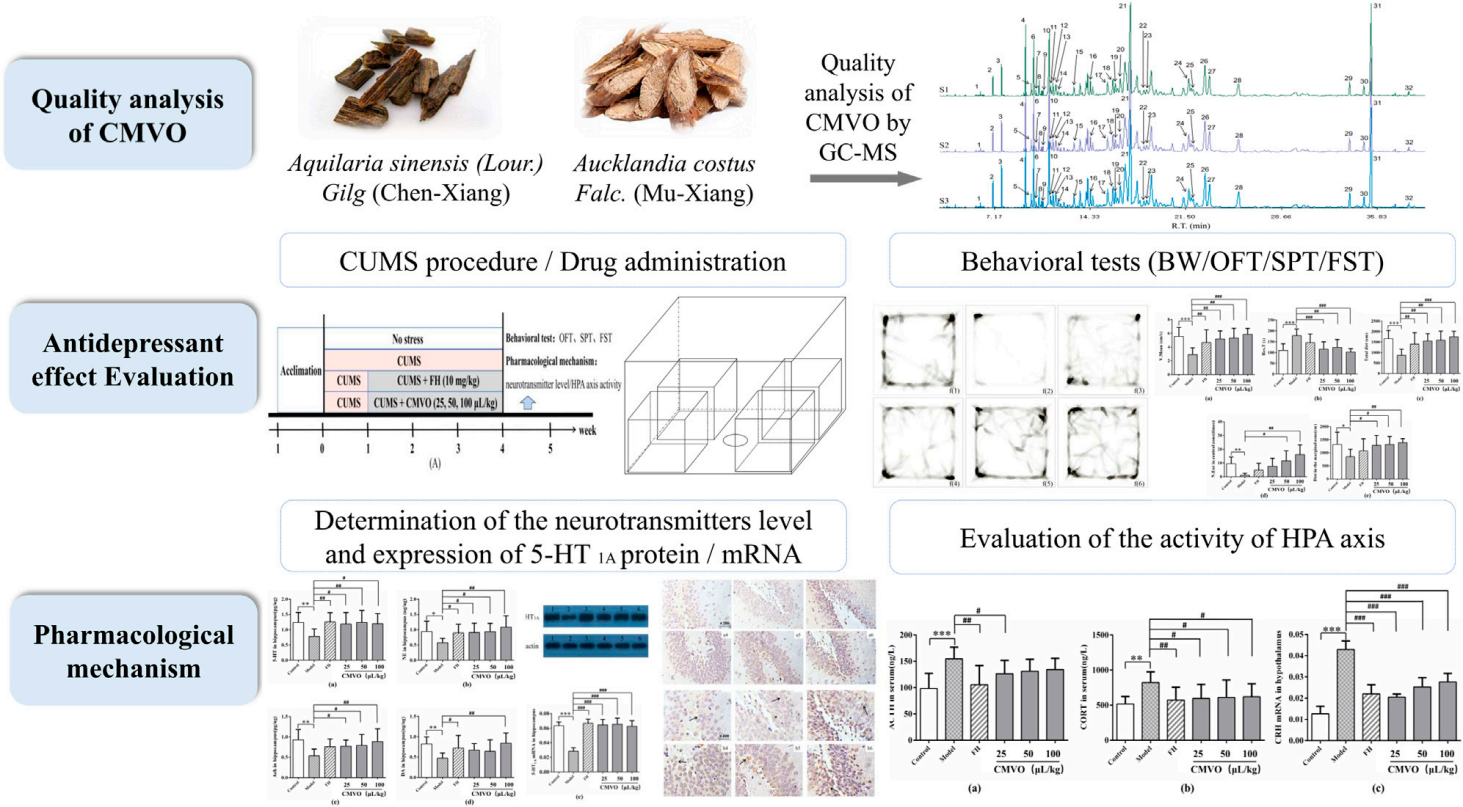
A schematic diagram of the research.

The antidepressant effect of CMVO *in vivo* was therefore investigated in this study. The CUMS depression model is recognized as reliable and practical, and is widely used to study the mechanism of depression (Willner, 1997; Zhuo et al., 2020). Accordingly, body weight assessment, sucrose preference test (SPT), forced swimming test (FST) and open field test (OFT) were executed in CUMS-induced rats to analyze the antidepressant effects of CMVO after inhalational administration. Previously, it was shown that CX volatile oil exerts an antidepressant effect by regulating hyperactivity of the HPA axis (Wang et al., 2018b), while the main active component of MX volatile oil exerts a neuroprotective effect on the hippocampus (Zhao et al., 2018). In addition, abnormal function of the HPA axis and deficiency of hippocampal monoamine neurotransmitters have been implicated in the pathogenesis of depression (Chen et al., 2019; Xing et al., 2019). Therefore, the possible effects of CMVO inhalational administration on depression were studied from the perspective of regulation of the HPA axis and neurotransmitters levels. The technical strategy of this study is shown in Figure 1.

## Materials and Methods

### Animals

All experimental procedures were approved by the Animal Ethics Committee of Jiangxi University of TCM. All efforts were made to minimize suffering and the number of animals used to produce reliable data.

Male Sprague Dawley rats weighing 180–220 g were supplied by Hunan Shrek Jingda Experimental Animal Co., Ltd. (Animal license No.: SCXK (Xiang) 2019-0004). The rats were housed at a constant temperature (23 ± 2°C), maintained on a 12 h light/dark cycle (lights on 8:00–20:00) and had free access to food and water. All rats were acclimatized for 7 days before the experiment.

### Materials and Reagents

The volatile oil of CX was obtained from Guangzhou Aroma Master Chenxiang Technology Co., Ltd. (Guangzhou, China) and the volatile oil of MX was obtained from Changshengyuan Medicinal Materials Development Co., Ltd. (Mianyang, China). Fluoxetine hydrochloride (FH) was purchased from Lilly Suzhou Pharmaceutical Co., Ltd. (Suzhou, China). The commercial enzyme-linked immunosorbent assay (ELISA) kits for adrenocorticotropic hormone (ACTH), corticosterone (CORT), 5-hydroxytryptamine (5-HT), dopamine (DA), norepinephrine (NE) and acetylcholine (Ach) were purchased from Jiangsu Enzymatic Immunity Industry Co., Ltd. (Yancheng, China). β-Actin and 5-HT_1A_ antibodies were purchased from Abcam (Cambridge, UK). The reagents (i.e., ethanol, chloroform, isopropanol, H_2_O_2_ and xylene) were of analytical reagent grade and were obtained from Sinopharm Chemical Reagent Co., Ltd. (Shanghai, China). Horseradish peroxidase (HRP)-conjugated secondary antibodies were obtained from Biyuntian Biotechnology Co., Ltd. (Shanghai, China). The SYBR Green PCR kit was purchased from Thermo Fisher Scientific (China) Co., Ltd. Neutral gum and phosphate buffered saline (PBS) solution were obtained from Beijing Solarbio Science & Technology Co., Ltd. (Beijing, China).

### Quality Analysis of ChenXiang-MuXiang Volatile Oil

Gas chromatography-mass spectrometry (GC-MS) was used to control the quality of CMVO. The gas chromatographic conditions were as follows: an Agilent HP-5MS (30 m × 250 µm × 0.25 µm) capillary column was used, the carrier gas was high purity He (99.999%), the sample volume was l µL, the shunt ratio was 10:1, and the flow rate was 1 mL/min. The temperature program was as follows: an initial temperature of 70°C, increased by 15°C/min up to 110°C, increased by 7°C/min up to 154°C (held for 5 min), increased by 1°C/min up to 155°C (held for 5 min), increased by 1°C/min up to 157°C (held for 5 min), increased by 2°C/min up to 165°C, and then increased by 10°C/min up to 300°C (held for 5 min). The mass spectrometry conditions were as follows: an EI ion source, the electron energy was 70 eV, the ion source temperature was 230°C, the MS quadrupole temperature was 150°C, the interface temperature was 250°C, the solvent delay was 3.0 min, the quality scan pattern was full scan, and the scan range was 30–650 amu. The chemical components were identified using the NIST 17.0 mass spectrometry database. The retention index (relative to C7-C40 n-alkanes, under the same gas chromatographic conditions) of each compound was calculated and compared with literature values to verify each compound identity.

### Chronic Unpredictable Mild Stress Procedure

The CUMS procedure was conducted as previously described (Willner, 1997; Dong et al., 2014) with minor modification. The rats in the control group were housed without interference, and were given food and water normally, except that water was withheld for 24 h before the SPT. The CUMS-induced rats were isolated in individual cages and randomly exposed to various stressors: restraint stress for 45 min, 1.5 min of tail nip (0.5–1 cm from the end of the tail), swimming in cold (4°C) water for 5 min, swimming in hot (40°C) water for 5 min, strange object stimulation, inversion of light/dark cycle for 24 h, food deprivation for 24 h, water deprivation for 24 h, level shaking for 5 min (1 time/s), wet bedding for 24 h (200 mL of water per individual cage to make the bedding wet), odor stimulation (ammonia or acetic acid) or cage tilting for 8 h (45°). The 12 types of stimulation (1 type per day) were applied randomly. Each stimulus appeared discontinuously, and the animals could not predict the occurrence of the stimulation, which lasted for 28 days.

### Drug Administration and Treatment

Before the modeling, the body weight and sucrose preference of each rat were tested. According to the results, the rats were divided into 6 groups and ensured that there was no significant difference in body weight and sucrose preference among all groups. The six groups are as follows: control group, CUMS model group, FH group (10 mg/kg), CMVO low, middle and high dose groups (25, 50, 100 μL/kg), with eight rats in each group. The main body of the aromatherapy inhalational administration device consists of a large transparent plexiglass box (measures 80 × 80 × 65 cm, covered) and four small transparent plexiglass boxes (measuring 30 × 30 × 30 cm). The small boxes were evenly placed at the bottom of the large box, and the aromatherapy atomizer that holds the volatile oil was placed in the middle of the large box. The CMVO was diluted into 10 ml of distilled water and was released into the glass box in the form of spray through an atomizer.

**FIGURE 2:**
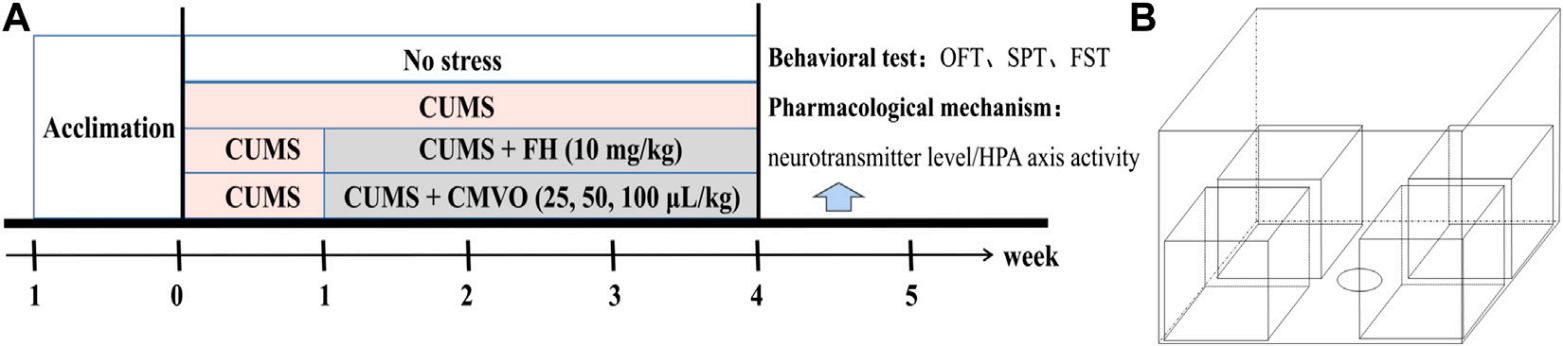
**(A)** Schematic representation of the experimental procedure. **(B)** The aromatherapy box used for inhaling administration.

All rats were subjected to CUMS for 1 week (except the blank group), and then the CMVO groups were given aromatherapy for 1 h every day for 3 weeks with the continued CUMS procedure. The control and CUMS groups were given 0.9% saline every day, and the FH group received intragastric administration of FH at a dose of 10 mg/kg, for 3 weeks. The experimental procedure is shown in Figure 2A and the aromatherapy box used for administration is shown in Figure 2B.

### Behavioral Tests

Depression-like behavior in rats was determined using the body weight (BW) test, OFT, SPT and FST.

#### Sucrose Preference Test and Body Weight

The SPT was carried out with reference to the previous literature (Daodee et al., 2019). The rats were trained to adapt to sucrose solution (1%, w/v) before the test. Two bottles of 1% sugar water were placed in each cage, and one of them was replaced with pure water 24 h later. After adaption, the rats were deprived of water and food for 24 h. Animals were then kept in separate cages with free access to two bottles, one filled with 200 mL sucrose solution (1%, w/v) and the other with 200 mL water. The positions of the bottles were balanced, and the two bottles were interchanged after 30 min to avoid side preference. The test was finished 1 h later and the sucrose preference was calculated by the following formula: sucrose preference (%) = sucrose consumption/(water consumption + sucrose consumption). All rats were weighed before and after treatment, and the BW change was assessed from the week-4 weight minus week-0 weight.

#### Open Field Test

The OFT was carried out with reference to the previous literature (Yi et al., 2013) with slight modification. The experiment was carried out in a square open black box. The laboratory was kept quiet, with constant light, a room temperature of 24 ± 1°C and humidity of 60–70%. A camera was installed directly above the central area of the black box. The rats were placed in the middle of the open field to explore freely, and the video analysis system was immediately activated to capture video automatically. The measuring time was 5 min for each rat, and the environment in the box was cleared after the end of measurement. The single-subject tracking mode of the system was used to measure average speed (V.mean), rest time (Res.T), number of times to enter the central area (N.Ent), total moving distance (Total dist) and Dist in the marginal zone of each rat.

#### Forced Swimming Test

The FST was carried out with reference to the previous literature (Wang et al., 2019a). The rats were placed in a cylinder with a height of 46 cm, a diameter of 45 cm, a depth of 41 cm, and a water temperature of 24 ± 1°C. The rats were exposed to a pre-test for 15 min, and the next day underwent the FST. The immobility time of the rats was observed for 5 min. The immobility time refers to the time it takes for the rats to float in the water without struggling, but only to keep their heads above the surface.

### Tissue Preparation

After 28 days of administration, the rats were killed and serum and brain tissue were collected. Blood was collected from the femoral artery, and the serum was separated by centrifugation after 30 min (3500 r/min centrifugation for 10 min at 4°C), and then stored in a refrigerator at −80°C. The hippocampus and hypothalamus of each rat were removed onto ice and stored in a refrigerator at −80°C after freezing with liquid nitrogen.

### Quantification of 5-Hydroxytryptamine, Dopamine, Norepinephrine, Acetylcholine in Hippocampus, and Adrenocorticotropic Hormone and Corticosterone in Serum by Enzyme-Linked Immunosorbent Assay

The rat hippocampi were used to determine the levels of neurotransmitters associated with CUMS-induced depression-like behavior. The serum was used to evaluate activity of the HPA axis associated with CUMS-induced depression-like behavior. The levels of 5-HT, DA, NE and ACh in hippocampus and the levels of ACTH and CORT in serum of rats were measured using ELISA kits. The operation was strictly in accordance with the corresponding instructions, and the concentration was normalized according to the standard curve.

### Detection of 5-HT_1A_ Content in Hippocampus by Western Blot

The total protein was extracted from fresh hippocampal tissue. A sample (20 µg) was separated by electrophoresis and transferred onto polyvinylidene difluoride membranes. After blocking with 5% skimmed milk powder at room temperature for 1 h, the membrane was incubated with primary antibody (5-HT_1A_, 1:1,000) at 4°C overnight, rinsed three times with tris-buffered saline/Tween 20 (TBST) for 5 min, and incubated with HRP-conjugated secondary antibody (1:1,000) for 1 h at 37°C. The membrane was rinsed with TBST for 5 min three times, and then analyzed using a chemiluminescence imaging system. Relative expression of protein = grayscale of target protein band/grayscale of β-actin band. The mean value of the intensity was obtained from three independent experiments.

### Detection of 5-HT_1A_ mRNA in Hippocampus and Corticotropin-Releasing Hormone mRNA in Hypothalamus by Reverse Transcription-Polymerase Chain Reaction

The RT-PCR reaction was conducted according to the SYBR Green PCR kit instructions. The expression levels of 5-HT_1A_ mRNA in hippocampus and CRH mRNA in hypothalamus were determined. The sequences of the primers used for real-time PCR are shown in Table 1. The set amplification procedure was as follows: 95°C, 10 min (95°C, 15 s; 55°C, 45 s) × 40; 95°C, 15 s; 60°C, 1 min; 95°C, 15 s; 60°C, 15 s. The data were analyzed by ABI Prism 7300 SDS software and the expression levels of 5-HT_1A_ and CRH mRNA were determined.

**TABLE 1:** Primer information for the RT-PCR experiment.

Gene name	Primer sequence (5’ → 3’)
5-HT_1A_	Primer F: TCT​CGC​TCA​CTT​GGC​TCA​TTG
	Primer R: TCC​TGA​CAG​TCT​TGC​GGA​TTC
CRH	Primer F: CTC​ACC​TTC​CAC​CTT​CTG​AG
	Primer R: GGCCAAGCGCAACATTTC
β-actin	Primer F: CGG​TCA​GGT​CAT​CAC​TAT​C
	Primer R: CAG​GGC​AGT​AAT​CTC​CTT​C

### Immunohistochemical Detection of 5-HT_1A_ in Hippocampus

The tissues were embedded, fixed and made into sections. Sections were deparaffinized and then hydrated with different concentrations of alcohol in double distilled water. The antigen was retrieved in 0.01 M trisodium citrate buffer and washed with 0.02 M PBS. The sections were incubated with 3% H_2_O_2_ for 10 min. Non-immune and normal goat serum were added to block non-specific antigen, followed by incubation in a wet box for 30 min at 37°C. The primary antibody was added and incubated overnight at 4°C. The sections were then incubated with HRP-conjugated secondary antibody (goat-anti rabbit) for 1 h at 37°C and visualized with 3,3′-diaminobenzidine. After washing, the sections were counterstained with hematoxylin, differentiated with 0.1% hydrochloric acid-alcohol and observed under a microscope to control the degree of staining. Finally, the sections were dehydrated in alcohol solution at different concentrations, cleared with xylene and sealed with neutral gum. A DM2500B microscope (Leica Corp., Wetzlar, Germany) was used to capture images and analyze the samples.

### Statistical Analysis

The experimental data were expressed as mean ± standard error of the mean (SEM) (x ± s). The experimental data were statistically analyzed and plotted with SPSS 21.0 (SPSS Inc., Chicago, IL, United States) and GraphPad Prism 6 software (GraphPad Software Inc., San Diego, CA, United States). One-way analysis of variance (ANOVA) was used for comparison between groups, LSD method was used for homogeneity of variance, Games-Howell method was used for heterogeneity of variance, where *p* < 0.05 indicated a significant difference and *p* < 0.01 indicated a very significant difference.

## Results

### Composition Analysis of ChenXiang-MuXiang Volatile Oil

**FIGURE 3:**
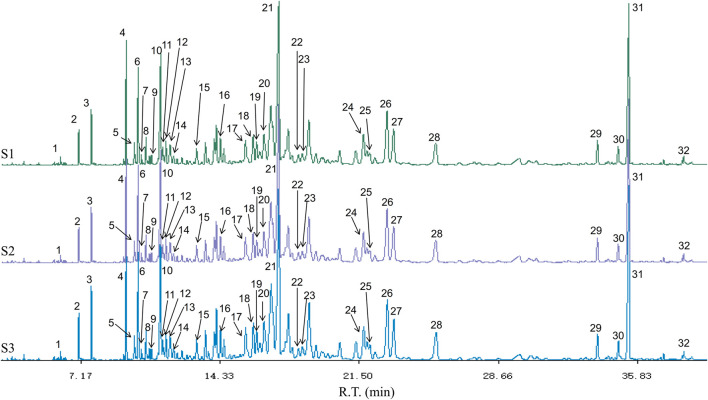
The GC-MS chromatogram of CMVO. S1-S3 represents three GC-MS chromatograms of CMVO samples, and three times GC-MS analysis were carried out to show the repeatability. The 32 components identified are numbered in the figure, which matches the serial numbers of each component in Table 2.

The components of CMVO were determined by GC-MS. Finally, 32 chemical constituents were identified. The GC-MS chromatogram and the 32 components are shown in Table 2 and Figure 3. The highest content of which is Aplotaxene (peak21, 13.3124%), followed by Dehydrocostus lactone (peak31, 8.5016%), β-Agarofuran (peak10, 5.4282%), Costol (peak26, 5.1089%), (-)-α-Costol (peak27, 3.2934%), etc. The GC-MS detection results of CMVO were compared with the previous studies, and it was found that Dehydrocostus lactone (peak31), Aplotaxene (peak21), Terpinen-4-ol (peak1), trans-a-Bergamotene (peak7) were the main characteristic components of the MX volatile oil (Yan et al., 2020), while Benzylacetone (peak2) (Miyoshi et al., 2013), β-Agarofuran (peak10), Agarospirol (peak19) were the unique components of CX volatile oil (Monggoot et al., 2018).

**TABLE 2:** The components of CMVO.

Peak No.	Identification	RT (min)	RI^a^	RI^b^	Area pct
1	Terpinen-4-ol	6.1047	1215	1180	0.154
2	Benzylacetone	7.043	1279	1257	0.9063
3	Anethole	7.6871	1321	/	1.4285
4	β-Elemen	9.4867	1430	1394	3.0074
5	Dihydro-α-ionone	9.9068	1452	1417	0.6224
6	Caryophyllene	10.0958	1462	1446	2.9496
7	trans-α-Bergamotene	10.2709	1471	1433	0.2446
8	Geranylacetone	10.502	1484	/	0.7454
9	Humulene	10.789	1499	1464	0.2634
10	β-Agarofuran	11.2582	1517	/	5.4282
11	β-Ionone	11.3772	1521	1516	0.5671
12	(+)-β-Selinene	11.5382	1527	1509	0.8105
13	(-)-α-Selinene	11.7413	1535	1493	1.2873
14	(3R,5aR,9S,9aS)-2,2,5a,9-Tetramethyloctahydro-2H-3,9a-methanobenzo[b]oxepine	11.9374	1542	/	0.4209
15	α-Elemol	13.1137	1586	1546	0.9187
16	Caryophyllene oxide	14.346	1622	1588	1.1901
17	2-((2S,4aR)-4a,8-Dimethyl-1,2,3,4,4a,5,6,7-octahydronaphthalen-2-yl)propan-2-ol	15.6414	1655	/	2.0584
18	(+)-γ-Eudesmol	16.0405	1665	1633	2.0719
19	Agarospirol	16.2156	1669	1645	1.5116
20	8,8,9,9-Tetramethyl-3,4,5,6,7,8-hexahydro-2H-2,4a-methanonaphthalene	16.5866	1679	/	3.1942
21	Aplotaxene	17.3569	1698	/	13.3124
22	Aromandendrene	18.3652	1716	1826	0.7836
23	α-Costal	18.5682	1720	1767	0.9397
24	(+)-4,11(13)-Eudesmadien-12-ol	21.7261	1774	/	2.5602
25	Phenol,2-ethyl-4,5-dimethyl-	22.0412	1780	/	0.9532
26	Costol	22.9445	1795	1734	5.1089
27	(-)-α-Costol	23.2735	1801	/	3.2934
28	Dehydrofukinone	25.4511	1829	/	2.8459
29	Dihydrodehydrocostus lactone	33.7695	1972	/	1.1565
30	n-Hexadecanoic acid	34.8548	2000	1985	0.9052
31	Dehydrocostus lactone	35.3939	2026	2007	8.5016
32	9-Octadecenoic acid	38.2158	2267	/	0.2981

RI, retention indices; RT, retention time.

^a^ RI calculated from RTs in relation to those of a series C_7_-C_40_ of n-alkanes.

^b^ RI from the literatures (Feng et al., 2016; Chen, 2019; Han et al., 2019; Huang et al., 2018; Shang, 2018; Geng, 2020; Song et al., 2020; Sun et al., 2020).

### Effects of Inhaling ChenXiang-MuXiang Volatile Oil on Depression-like Behavior Induced by Chronic Unpredictable Mild Stress in Rats

The depression-like behavior in CUMS rats was assessed to examine the antidepressant effects of CMVO using the OFT, FST and SPT. The body weights of all rats before and after modeling were measured.

#### Effects of ChenXiang-MuXiang Volatile Oil on Body Weight and Sucrose Preference Test

**FIGURE 4:**
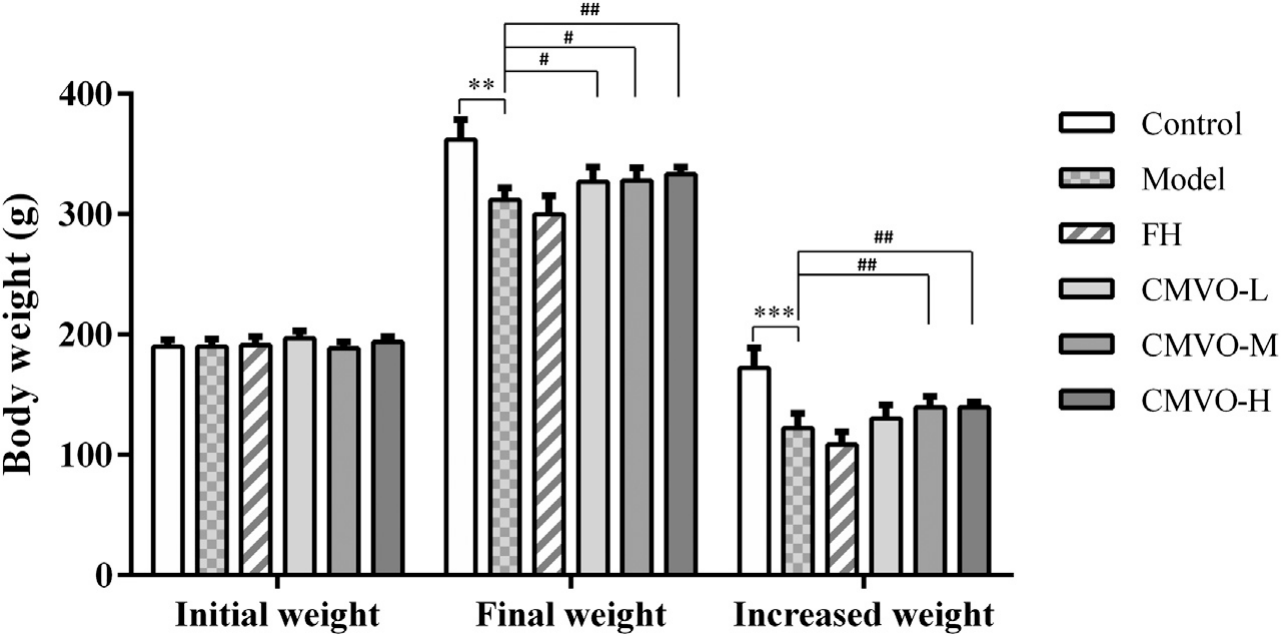
The BW in each group at weeks 0 and 4 (Mean ± SD, n = 8). The BW was recorded at weeks 0 and 4, and the increased weight is expressed as the BW at week 4 minus the BW at week 0. *p < 0.05, **p < 0.01, ***p < 0.001 vs. control group. #*p* < 0.05, ##*p* < 0.01, ###*p* < 0.001 vs. CUMS group.

Before the CUMS modeling process, there were no significant differences in the baseline BW, as shown in Figure 4. After treatment for 4 weeks, differences in BW showed statistical significance among the groups (F_5,42_ = 23.617, *p* < 0.01). Compared with the control group, the BW of the model group increased slowly and decreased significantly after modeling (*p* < 0.01), which suggests that CUMS may cause gastrointestinal dysfunction in rats, resulting in loss of appetite and individual growth retardation. Compared with the model group, the weights in the CMVO-L, CMVO-M and CMVO-H groups significantly increased (*p* < 0.05 or *p* < 0.01).

**FIGURE 5:**
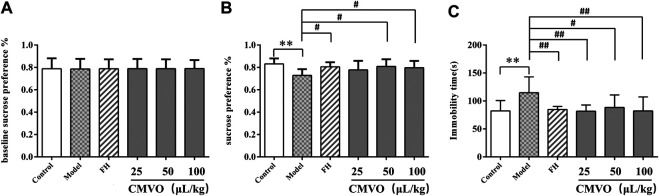
Effects of CUMS and CMVO treatment (25, 50, 100 μL/kg) on baseline sucrose preference **(A)**, SPT **(B)** and FST **(C)**. Values are expressed as (Mean ± SD, n = 8). *p < 0.05, **p < 0.01, ***p < 0.001 vs. control group. #*p* < 0.05, ##*p* < 0.01, ###*p* < 0.001 vs. CUMS group.

Before the modeling process, there were no significant differences in the baseline sucrose preference, as shown in Figure 5A. In the SPT (Figure 5B), the results showed that there were significant differences in sucrose preference among different groups (F_5,42_ = 2.777, *p* < 0.05). Compared with the control group, the sucrose preference rate in the model group decreased significantly (*p* < 0.01). Compared with the model group, the sucrose preference rate of other groups increased, and there were significant differences in the fluoxetine hydrochloride (FH), CMVO-M and CMVO-H groups (*p* < 0.05).

#### Effects of ChenXiang-MuXiang Volatile Oil on the Immobility Time in Forced Swimming Test

The effect of CMVO on FST in CUMS rats is shown in Figure 5C. There were significant differences in immobility time among the groups (F_5,42_ = 3.281, *p* < 0.05). The immobility time during forced swimming in the model group was significantly increased compared with that in the control group (*p* < 0.01). In the FST, lower immobility time was observed in FH- and CMVO-treated CUMS rats, and there were significant differences in the FH, CMVO-L and CMVO-H groups (*p* < 0.01), and in the CMVO-M group (*p* < 0.05).

#### Effects of ChenXiang-MuXiang Volatile Oil on Open Field Test

**FIGURE 6:**
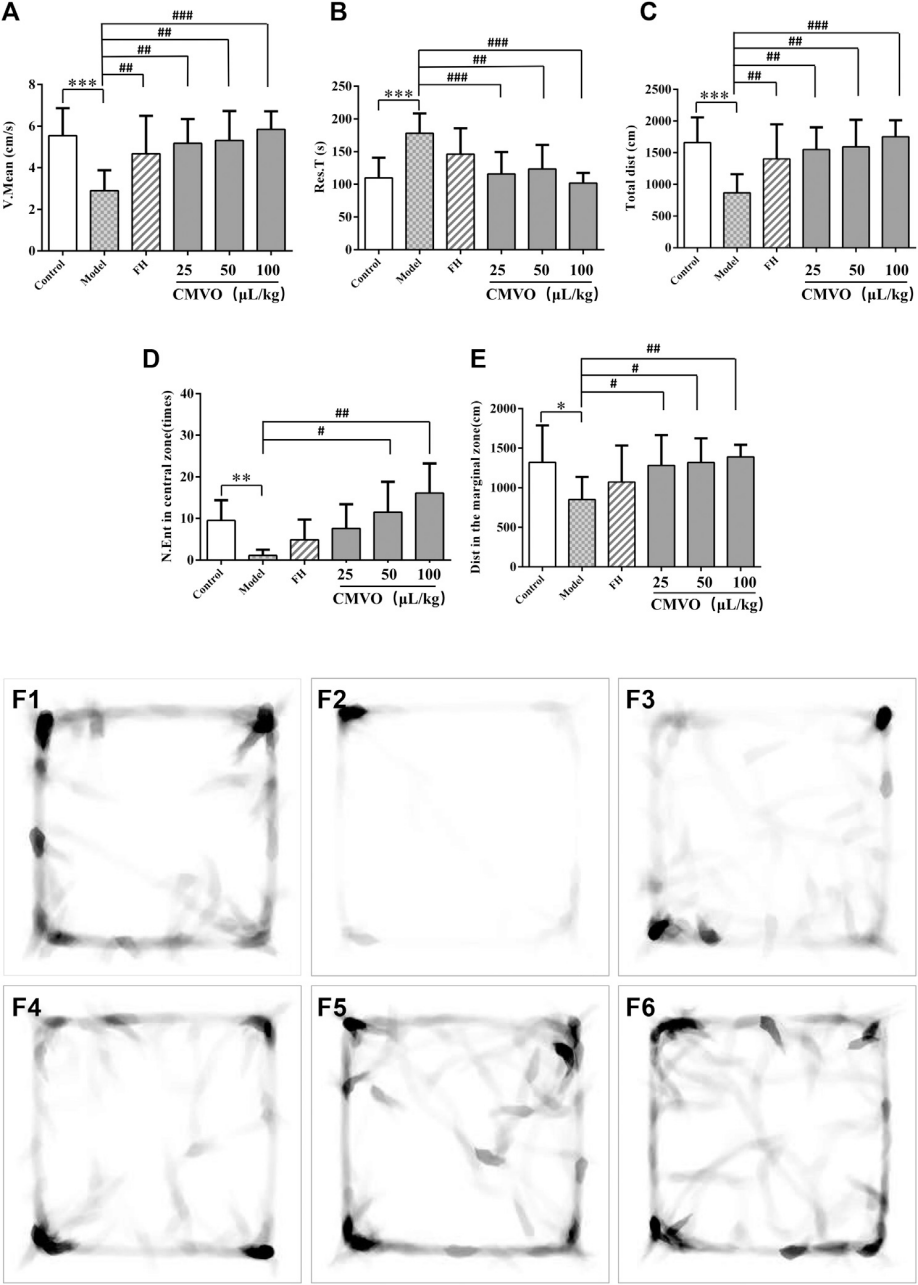
The V.mean, Res.T, Total dist, N.Ent in central zone and Dist in the marginal zone of rats on OFT **(A,B,C,D,E)** and the heat map (F1‐F6) of the rat’s movement trajectory for 5 min in the open field (f1-f6). Values are expressed as (Mean ± SD, n = 8). *p < 0.05, **p < 0.01, ***p < 0.001 vs. control group. #*p* < 0.05, ##*p* < 0.01, ###*p* < 0.001 vs. CUMS group.

Figures 6A–E show the effects of CMVO on the OFT in CUMS rats. The results of one-way ANOVA showed that there were significant differences in V.mean, Dist, Res.T, N.Ent and Dist in the marginal zone among the groups (F_5,42_ = 5.289, *p* < 0.01; F_5,42_ = 5.314, *p* < 0.01; F_5,42_ = 6.295, *p* < 0.01; F_5,42_ = 7.006, *p* < 0.01; F_5,42_ = 5.314, *p* < 0.01). Compared with the control group, V.mean, Total Dist, Dist in the marginal zone and N.Ent decreased significantly (*p* < 0.05 or *p* < 0.01), and Res.T increased significantly (*p* < 0.001), which indicated that the activity and curiosity about the novel environment of the model group rats were decreased (Cao et al., 2019b). Compared with the model group, V.mean and Dist in the FH group and all CMVO dose groups increased significantly (*p* < 0.01), while Res.T in all CMVO dose groups decreased significantly (*p* < 0.01) and N.Ent in CMVO‐M and CMVO‐H groups increased significantly (*p* < 0.05 or *p* < 0.01). This showed that the willingness of rats in the treatment groups to explore the environment was enhanced, indicating that CMVO exerted an antidepressant effect. Figures 6F (1–6) show heat maps of the rat’s movement trajectory for 5 min in the open field.

### Effect on Hypothalamic–Pituitary–Adrenal Axis Activity in Rats

In order to observe the regulatory effect of CMVO on the HPA axis in CUMS rats, the levels of ACTH and CORT in serum were determined by ELISA and the expression level of CRH mRNA in hypothalamus was measured by RT-PCR. Significant differences among the groups were observed on ACTH and CORT levels in serum, and CRH mRNA expression level in hypothalamus (F_5,42_ = 4.715, *p* < 0.01; F_5,42_ = 2.572, *p* < 0.05; F_5,12_ = 21.467, *p* < 0.01).

**FIGURE 7:**
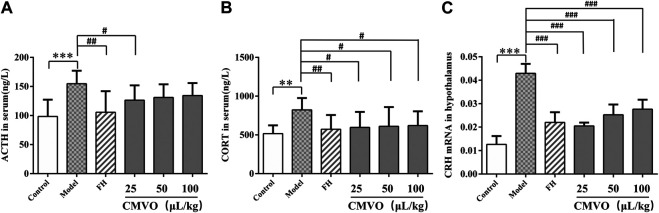
Effects of CMVO treatment on ACTH **(A)** and CORT **(B)** levels (n = 8) and CRH mRNA expression **(C)** (n = 3). The levels of ACTH and CORT in the hippocampus of rats were tested by ELISA, and the CRH mRNA in the hypothalamus was tested by RT-PCR. Values are expressed as (Mean ± SD). *p < 0.05, **p < 0.01, ***p < 0.001 vs. control group. #*p* < 0.05, ##*p* < 0.01, ###*p* < 0.001 vs. CUMS group.

The serum levels of ACTH and CORT in the CUMS group were significantly higher than those in the control group (*p* < 0.01). Compared with the model group, treatment with FH and CMVO remarkably decreased ACTH levels. There were significant differences in the FH group (*p* < 0.01) and CMVO-L group (*p* < 0.05). In addition, FH or CMVO treatment also reversed the increase of serum CORT induced by CUMS. There were significant differences in the FH and all CMVO dose groups (*p* < 0.01 or *p* < 0.05). The RT-PCR results showed that CUMS significantly increased hypothalamus expression of CRH mRNA in the model group (*p* < 0.001). The expression of CRH mRNA decreased significantly after treatment with FH, CMVO-L, CMVO-M and CMVO-H compared with the model group (*p* < 0.001). The results are shown in Figures 7A–C. These results suggest that CUMS can lead to overexcitation of the HPA axis, increasing expression of ACTH, CORT and CRH mRNA in rats. The overexcitation of the HPA axis was regulated by inhalation of CMVO, which may be a mechanism for the action of CMVO in the treatment of depression.

### Effects on the Levels of Neurotransmitters (5-Hydroxytryptamine, Norepinephrine, Acetylcholine and Dopamine)

**FIGURE 8:**
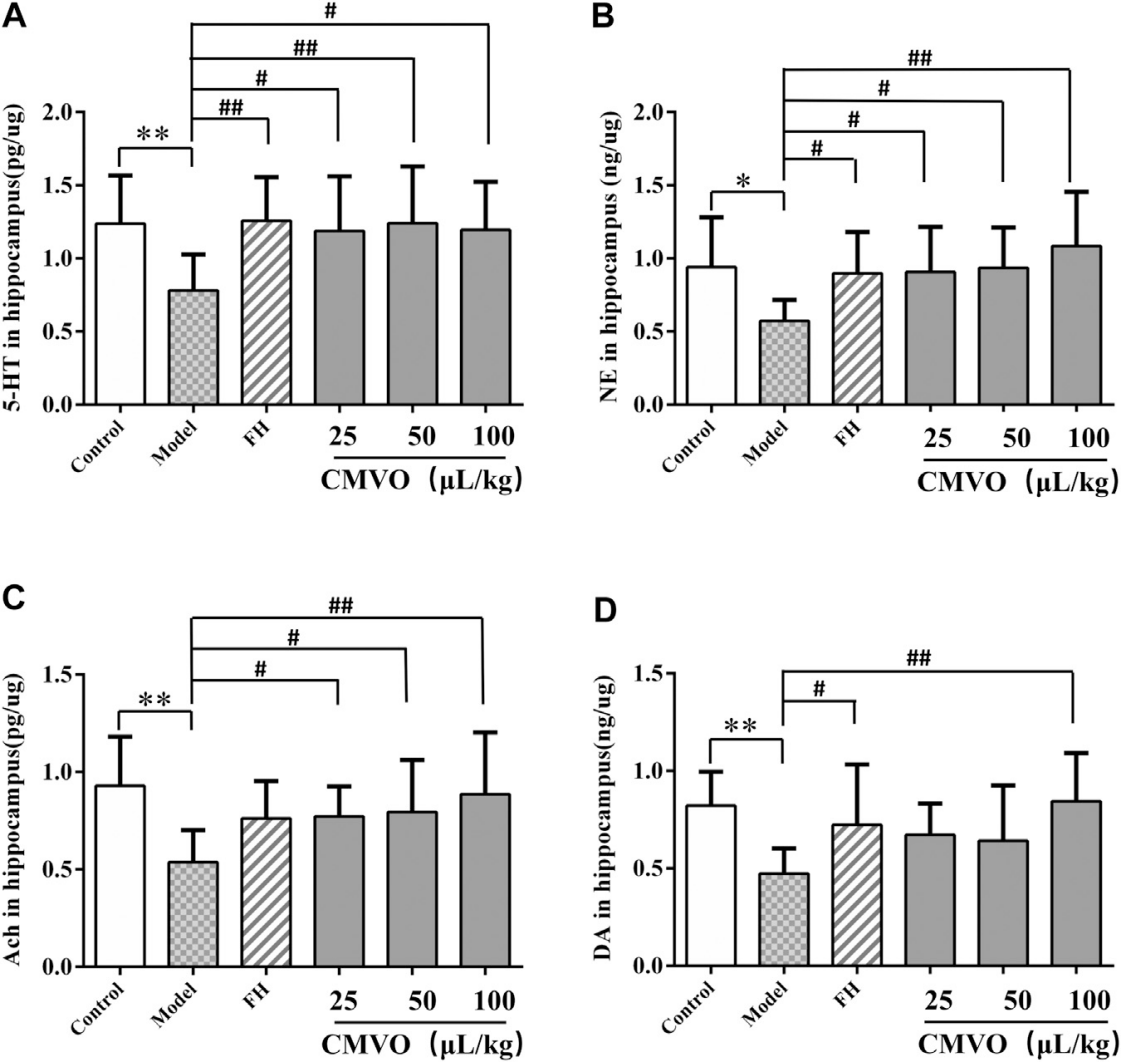
Effects of CMVO treatment on 5-HT, NE, Ach and DA levels in hippocampus **(A,B,C,D)**. The levels of 5-HT, NE, Ach and DA in the hippocampus of rats were tested by ELISA. Values are expressed as (Mean ± SD, n = 8). *p < 0.05, **p < 0.01, ***p < 0.001 vs. control group. #*p* < 0.05, ##*p* < 0.01, ###*p* < 0.001 vs. CUMS group.

The levels of neurotransmitters in the hippocampus were assessed to reveal the role of CMVO in treatment of depression. Significant effects were observed on 5-HT, NE, ACh and DA in the hippocampus (F_5,42_ = 2.449, *p* < 0.05; F_5,42_ = 2.636, *p* < 0.05; F_5,42_ = 2.781, *p* < 0.05; F_5,42_ = 2.855, *p* < 0.05). The levels of 5-HT, NE, ACh and DA in the hippocampus of CUMS-induced rats were significantly decreased compared with the control group (*p* < 0.01 or *p* < 0.05) (Figures 8A–D and Table 3). Treatment with CMVO reversed the decrease of neurotransmitters induced by the CUMS procedure. Compared with the model group, the levels of 5-HT in the FH, CMVO-L, CMVO-M and CMVO-H groups were significantly increased (*p* < 0.01 or *p* < 0.05). The levels of NE in each group were also increased by varying degrees, with significant differences in the FH, CMVO-L and CMVO-M groups (*p* < 0.05) and the CMVO-H group (*p* < 0.01). After treatment with FH and CMVO, the levels of ACh significantly increased compared with the model group, with significant differences in all CMVO dose groups (*p* < 0.01 or *p* < 0.05). Compared with the model group, the DA levels increased significantly in the FH group (*p* < 0.05) and CMVO-H group (*p* < 0.01).

**TABLE 3:** Contents of 5-HT,Ach,DA, NE in the hippocampus (x ± s, n = 8).

Groups	5-HT (pg/ug)	Ach (pg/ug)	DA (ng/ug)	NE (ng/ug)
Control group	1.24 ± 0.33	0.93 ± 0.25	0.82 ± 0.17	0.94 ± 0.34
CUMS group	0.78 ± 0.25**	0.54 ± 0.17**	0.47 ± 0.13**	0.57 ± 0.14*
FH group	1.26 ± 0.30##	0.76 ± 0.19	0.72 ± 0.31#	0.90 ± 0.28#
CMVO-L group	1.19 ± 0.37#	0.77 ± 0.15#	0.67 ± 0.16	0.91 ± 0.31#
CMVO-M group	1.24 ± 0.39##	0.79 ± 0.27#	0.64 ± 0.28	0.94 ± 0.28#
CMVO-H group	1.20 ± 0.33#	0.89 ± 0.32##	0.84 ± 0.25##	1.08 ± 0.37##

### Effect of ChenXiang-MuXiang Volatile Oil on the Level of 5-HT_1A_ Protein in the Hippocampus

**FIGURE 9:**
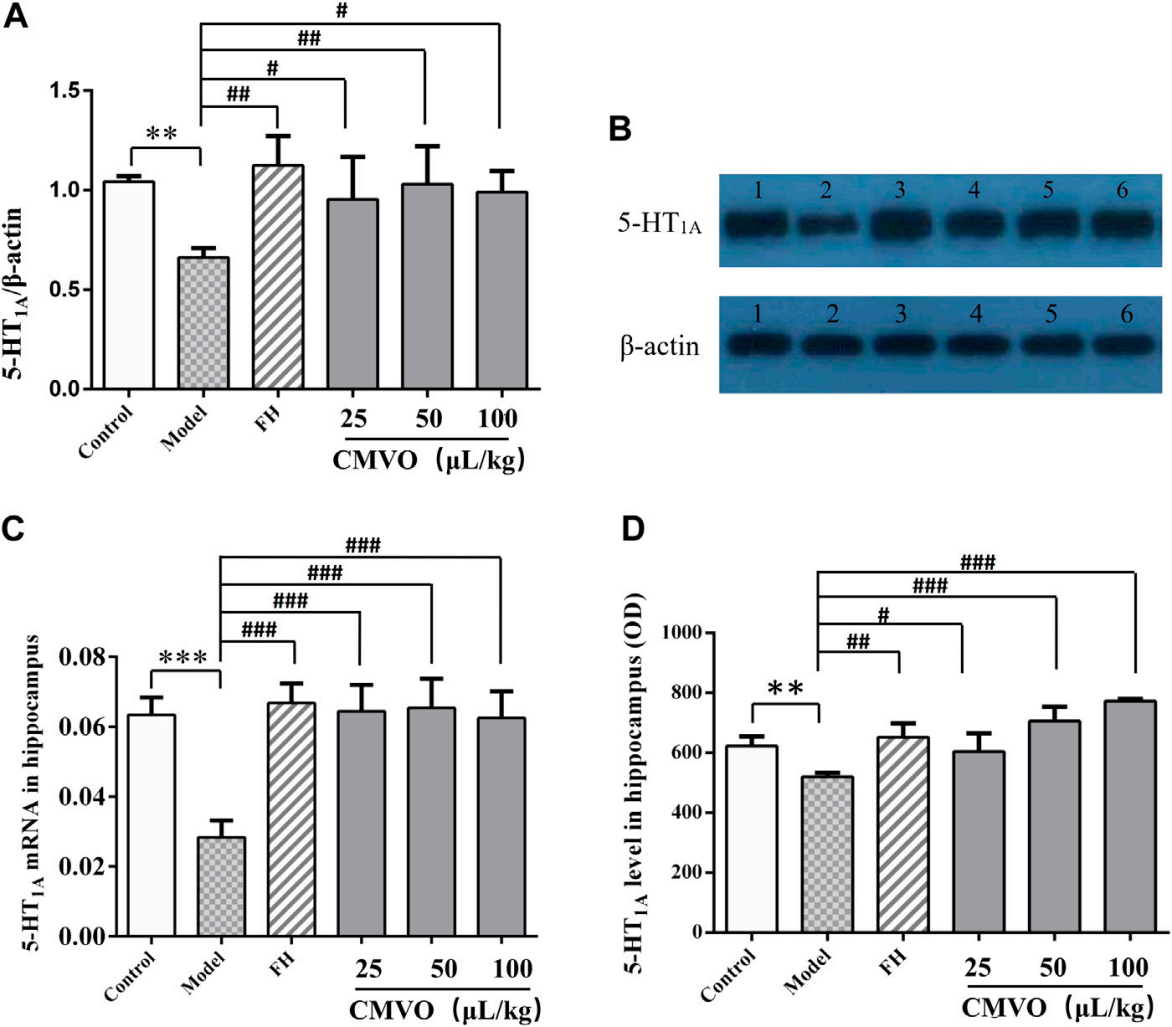
**(A,B)** Effects of CMVO on the level of 5-HT_1A_ protein in the hippocampus (Mean ± SD, n = 3). The results of Western blot are shown in Figures 9A,B (1:control group, 2:CUMS group, 3:FH group, 4:CMVO-L group, 5:CMVO-M group, 6: CMVO-H group). **(C)** Effects of CMVO on the level of 5-HT_1A_ mRNA in the hippocampus (Mean ± SD, n = 3). The results of the RT-PCR are shown in Figure 9C. (Mean ± SD, n = 3). **(D)** Effect of CMVO on the expression of 5-HT_1A_ in the hippocampus by immunohistochemistry (Mean ± SD, n = 3). The results of immunohistochemical staining are shown in Figure 9D (OD: optical density). Values are expressed as mean ± SEM. *p < 0.05, **p < 0.01, ***p < 0.001 vs. control group. #*p* < 0.05, ##*p* < 0.01, ###*p* < 0.001 vs. CUMS group.

The expression level of 5-HT_1A_ in hippocampus of rats was semi-quantitatively analyzed by calculating the ratio of the gray value of 5-HT_1A_ bands to β-actin bands. The results showed that 5-HT_1A_ gene transcription in hippocampus was significantly decreased in the CUMS group compared with the control group (F_5,12_ = 3.953, *p* < 0.01) (Figures 9A,B). The expression of 5-HT_1A_ was significantly increased in the FH and CMVO-M groups (*p* < 0.01), CMVO-L and CMVO-H groups (*p* < 0.05) compared with the CUMS group.

### Effect of ChenXiang-MuXiang Volatile Oil on the Expression of 5-HT_1A_ mRNA in the Hippocampus

Similarly, RT-PCR analysis showed that expression of 5-HT_1A_ mRNA in the hippocampus of the model group was significantly decreased compared with the control group (*p* < 0.001), while the expression of 5-HT_1A_ mRNA in the CMVO treatment groups was significantly reversed (*p* < 0.001) (Figure 9C).

### Effect of ChenXiang-MuXiang Volatile Oil on the Expression of 5-HT_1A_ in the Hippocampus

**FIGURE 10:**
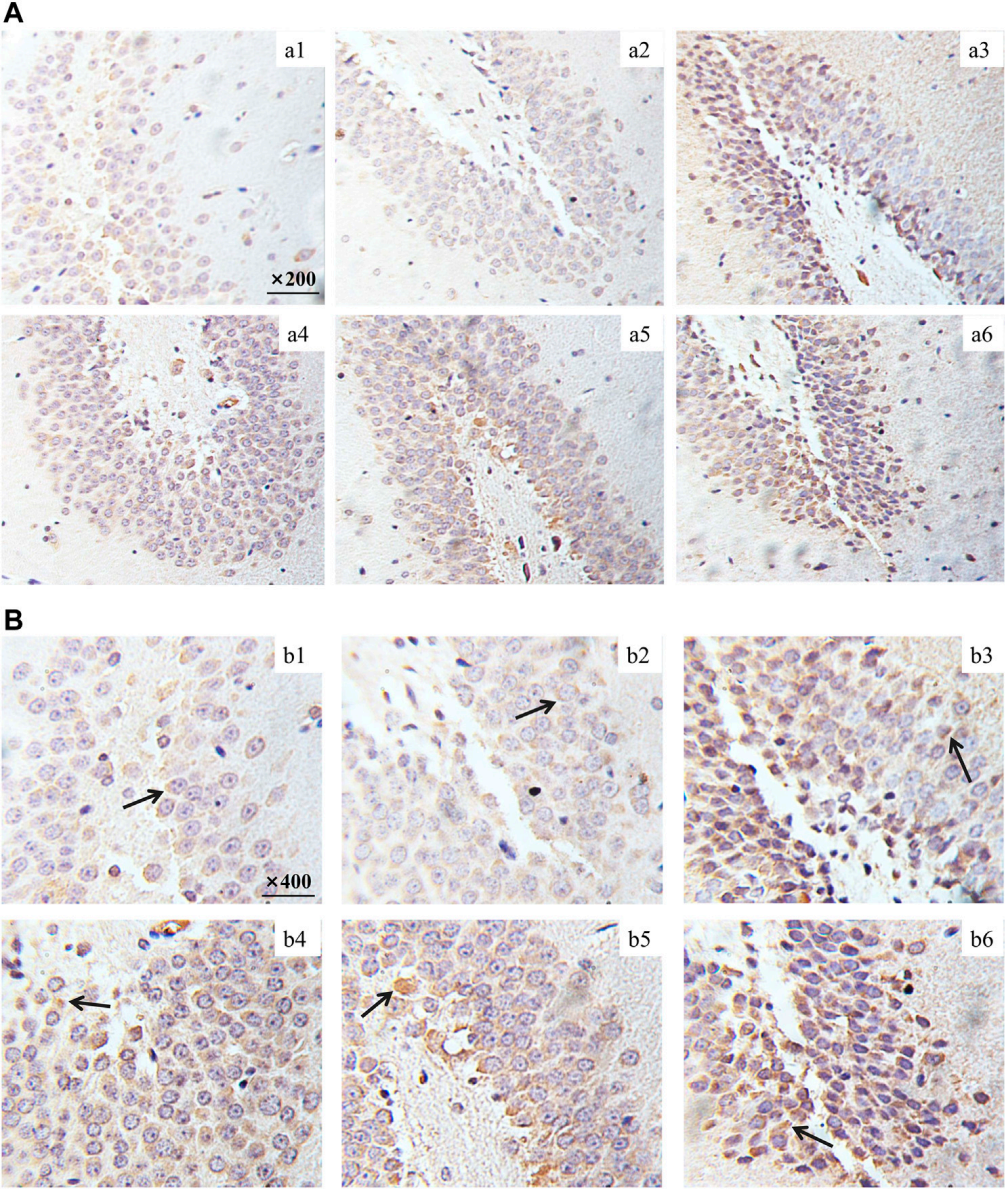
Effect of CMVO on the expression of 5-HT_1A_ in the hippocampus. The immunohistochemical staining pictures taken by microscope are shown in Figure 10A (Magnification:×200) and Figure 10B (Magnification:×400).

5-HT_1A_ positive cells in the hippocampus of each group were examined by immunohistochemistry (Figures 9D, 10A,10B). The OD value of the 5-HT_1A_ positive cells in the hippocampus was analyzed (Figure 9D). Compared with the control group, the expression of 5-HT_1A_ positive cells in the CUMS group was significantly reduced (*p* < 0.01). Compared with the CUMS group, the expression of 5-HT_1A_-positive cells was significantly increased in the FH (*p* < 0.01) and CMVO groups (*p* < 0.05 or *p* < 0.001).

## Discussion

In the CUMS model, the state of reduced interest and lack of pleasure shown by animals is similar to the clinical symptoms of depression. Lack of pleasure is an important manifestation of depression and the SPT is widely used to evaluate the degree of pleasure deficiency in depressed animals (Xu et al., 2011). In this study, the sucrose preference index in the CUMS group decreased significantly, indicating that the response of animals to reward decreased and that the depression model was established successfully. The treatment with CMVO increased the sucrose preference index and showed an antidepressant effect. The efficacy of antidepressants is widely evaluated using FST, in which the immobility time reflects the degree of despair in the rats (Porsolt et al., 1978; Xing et al., 2015). Consistent with previous studies, CUMS significantly prolonged the immobility time in the FST, while CMVO administration shortened the immobility time. The OFT is based on the phenomenon that rats are afraid to enter the open or bright field to reflect their emotions, and depressive behavior is evaluated by the desire of the rats to explore in the open field (Xing et al., 2015). The desire to explore in the open field decreased in the model group, total moving distance and the number of times to enter the central area decreased, immobility time was prolonged and movement was slower. After administration of CMVO, these indexes were reversed. Analysis of BW, and OFT, FST and SPT results showed that the rats in the model group exhibited obvious depressive behavior, indicating successful establishment of the model. Treatment with different doses of CMVO reversed these depressive behaviors and showed an obvious antidepressant effect. Although there was an obvious antidepressant effect in the FH group according to the behavioral experiments (OFT, FST, SPT), the body weights of rats decreased slightly compared with the model group, which was not consistent with some experimental results reported previously (Xing et al., 2019; Li et al., 2020c). On the one hand, according to the literature (Willner, 1997), although the commonly used chronic antidepressant drugs can normalize sucrose intake, they may not reverse CUMS-induced weight loss (Willner et al., 1987). In the CUMS model, there is no obvious positive correlation between sucrose preference index and body weight, which is consistent with the report of the founder of the model (Willner, 1997; Zhang et al., 2018). On the other hand, the results showed that FH did not improve the appetite or digestive function of depressed rats in the short term, but that CMVO significantly improved the body weight of the rats, suggesting that CMVO may have a better effect on anorexia caused by depression.

Depression is a disease involving a variety of neurotransmitters and brain regions, and its pathogenesis has not been fully elucidated. However, it is generally recognized that chronic stress plays an important role in the development of depression. Long-term chronic stress can lead to dysfunction of the HPA axis and increased secretion of corticosteroids, both of which are considered to be closely involved in the pathogenesis of human depression (Jozuka et al., 2003; Xing et al., 2015). The HPA axis is one of the complex neurobiological mechanisms contributing to the occurrence and development of depression (Xing et al., 2015). During the stress process, CRH is released from the paraventricular nucleus of the hypothalamus and promotes the release of ACTH from the pituitary. This results in the release of glucocorticoids from the adrenal cortex, providing feedback to the HPA axis through the glucocorticoid receptor (Zhang et al., 2019). In patients suffering from depression, the activity of the HPA axis is abnormally increased. The concentrations of cortisol are generally increased, which eventually destroys the negative feedback regulation of the HPA axis. Therefore, the concentrations of ACTH and CORT in serum and the expression of CRH mRNA in the hypothalamus were measured after the CUMS procedure. It was found that ACTH and CORT levels and the expression of CRH mRNA were increased significantly in CUMS rats, which was consistent with published literature (Song et al., 2003; Li et al., 2020b) that chronic stress can activate the HPA axis. The administration of CMVO significantly decreased the levels of ACTH and CORT in the serum of CUMS rats. These results suggest that the inhibition of hyperfunction of the HPA axis and restoration of the negative feedback loop may be an important mechanism of the CMVO antidepressant effect.

Moreover, there is a close relationship between the HPA axis and neurotransmitters. Overexcitation of the HPA axis may damage monoaminergic neurons in the hippocampus, resulting in decreased release of monoamine neurotransmitters (Zhang et al., 2019). Studies proved that the disruption of negative feedback regulation of the HPA axis will further impair the function of the hippocampus, which is closely involved in memory and emotion, and eventually lead to lower levels of neurotransmitters and aggravate depression (Mahar et al., 2014; Cao, 2019a). Through inhaled administration, volatile compounds can bind to olfactory receptors, trigger electrophysiological responses, stimulate the brain and alleviate depression-like behavior (Park et al., 2015; Zhang et al., 2019). In addition, it may prevent excessive activation of the HPA axis and stimulate the brain to achieve antidepressant effects by increasing concentrations of neurotransmitters (Watanabe et al., 2011; Lv et al., 2013; Zhang et al., 2019).

The hippocampus plays an important role in emotional regulation and is an important region that mediates the stress response (Peng et al., 2015; Sheline et al., 2019). The causes of depression are thought to be associated with the serotonergic system, norepinephrine, and dopamine (Li et al., 2020c). The monoamine hypothesis is the first neurobiochemical theory of depression, which holds that deficiency of NE, 5-HT or DA in the synaptic space *in vivo* is the main cause of depression (Duffey et al., 2013). In fact, most of the commonly used antidepressants are 5-HT reuptake inhibitors. Anhedonia, the core clinical feature of depression, is most likely linked to abnormality of the DA-reward pathway (Chen et al., 2019). The imbalance between acetylcholine and adrenergic neurons may lead to depression (Janowsky et al., 1972), and studies have shown that ACh-NE signals can mediate behaviors related to anxiety and depression through the interaction between β2nAChRs and α2-norepinephrine receptors (Mineur et al., 2018; Meng et al., 2020). Therefore, we determined the levels of monoamine neurotransmitters (5-HT, NE and DA) and cholinergic neurotransmitter (ACh) in the hippocampus of rats. In the CUMS group, the levels of 5-HT, NE, DA and ACh were significantly decreased. Their levels were significantly increased after intervention with FH and CMVO, indicating that CMVO may exert an antidepressant effect by regulating the levels of neurotransmitters. The levels of ACh and NE in the CUMS model group decreased significantly, and both levels were significantly increased by CMVO intervention, indicating that CMVO can regulate the interaction of the ACh-NE signal. The 5-HT_1A_ receptor subtype is considered to be an important mediator for the stimulatory influence of 5-HT on stress HPA activity (Goel et al., 2014). The 5-HT_1A_ receptor is closely involved in emotional disorders, and its blockade can lead to spontaneous antidepressant behavior in mice (Stewart et al., 2014). In order to further explore the mechanism of CMVO, we evaluated the change of 5-HT_1A_ levels by immunohistochemistry, RT-PCR and western blot. The results showed that 5-HT_1A_ decreased significantly in the model group, and CMVO administration increased the level, indicating that CMVO may regulate levels of 5-HT by up-regulating the expression of 5-HT_1A_. The results of our study suggest that CMVO can regulate the metabolism of neurotransmitters in the hippocampus, which may underlie its mechanism of action, and its effect is similar to that of the established drug, fluoxetine hydrochloride.

32 components of CMVO were identified by GC-MS analysis, of which sesquiterpenes were the main components. We speculate that CMVO may play a comprehensive antidepressant effect through these components. In recent years, many literatures have reported the effects of the components in the CX and MX volatile oil on the central nervous system. At present, the most concerned compounds with potential antidepressant activity in CX essential oil are agarofuran-like derivatives, among which buagafuran is the most potential and phase II clinical trials are being conducted on it (Wang et al., 2018c). It’s potential mechanism might be through modulating central neurotransmitters, such as dopamine (Zhang et al., 2004). The CX volatile oil has definite sedative effect by inhalational administration, in which agarospirol (Okugawa et al., 1996; Okugawa et al., 2000), benzylacetone, α-gurjunene, and (+)-calarene are the main components (Takemoto et al., 2008). Study (Okugawa et al., 2000) have shown that agarospirol in CX essential oil and dehydrocostus lactone in MX essential oil were found to be the principle products evaluated as analgesics in pharmacological studies. In addtion, the two components has shown to be potent as an antagonist of dopamine D_2_ and serotonine 5-HT_2A_ receptor binding (Okugawa et al., 2000). Based on the above research results, it can be concluded that the components such as dehydrocostus lactone in MX volatile oil, β-Agarofuran, agarospirol and benzylacetone in CX volatile oil may have comprehensive effects on the central nervous system by regulating neurotransmitters such as 5-HT and DA. Similarly, our results also show that CMVO can regulate the level of neurotransmitters such as 5-HT and DA, thus playing an antidepressant effect. Besides, aplotaxene is the most abundant component in CMVO, study have shown that it has a good immune function, and has been proved to be a novel immunotherapeutic agent for immunological diseases related to the overactivation of T cells. On the other hand, study (Yamahara et al., 1990) have shown that sesquiterpenes such as β-eudesmol and hinesol can clearly enhanced intestinal charcoal transport in mice. It has been found that benzylacetone, the main active compound in the CX volatile oil, has the effect of boosting appetite (Okugawa et al., 2016a; Okugawa et al., 2016b). As mentioned in the experimental results, CMVO significantly increased the body weight of rats within the 21 days’ administration, showing a better effect compared with positive drug FH. Depression is common symptoms such as loss of appetite, and CMVO is likely to play a role in gastrointestinal regulation through these compounds. Symptoms such as loss of appetite are common in patients with depression, and CMVO is likely to improve gastrointestinal function through these components. To sum up, various components of CMVO may play antidepressant effect by regulating neurotransmitters’ levels, immunity and gastrointestinal function.

## Conclusion

In conclusion, this study demonstrated that CMVO inhalational administration exerts antidepressant effects in CUMS rats. Its potential mechanism of action is related to inhibition of the hyperactive HPA axis and regulation of monoamine neurotransmitter levels in the hippocampus. This study may provide medicines with definite curative effect and suitability for long-term administration in patients with depression. CX and MX are two classical aromatic traditional Chinese herbs which are frequently used in antidepressant prescriptions, but most of the studies have focused on the efficacy of their oral administration, and the antidepressant effects and mechanisms of the combination of the two volatile oils have not been studied. In this study, a self-made aromatherapy device was used to study the antidepressant effect and mechanism of CMVO through inhalational administration. At present, most of the antidepressants are oral drugs, and there are some deficiencies in the treatment of depression, a long-term chronic disease. This study explored a new administration method for CX-MX, a traditional herbal pair, to play an antidepressant effect, and it can also provide a reference for others to study the antidepressant effect of aromatic traditional Chinese herbs by inhalational administration.

## Data Availability

The original contributions presented in the study are included in the article, further inquiries can be directed to the corresponding authors.
